# Landscape genetics reveal broad and fine‐scale population structure due to landscape features and climate history in the northern leopard frog (*Rana pipiens*) in North Dakota

**DOI:** 10.1002/ece3.4745

**Published:** 2019-01-15

**Authors:** Justin M. Waraniak, Justin D. L. Fisher, Kevin Purcell, David M. Mushet, Craig A. Stockwell

**Affiliations:** ^1^ Department of Biological Sciences, Environmental and Conservation Sciences Graduate Program North Dakota State University Fargo North Dakota; ^2^ U.S. Geological Survey Northern Prairie Wildlife Research Center Jamestown North Dakota; ^3^ Present address: Natural Resource Conservation Service Fergus Falls Minnesota; ^4^ Present address: Data Science and Analytics Program Harrisburg University Harrisburg Pennsylvania

**Keywords:** amphibians, isolation by distance, paleoclimate, post‐Pleistocene recolonization, species distribution model, watershed

## Abstract

Prehistoric climate and landscape features play large roles structuring wildlife populations. The amphibians of the northern Great Plains of North America present an opportunity to investigate how these factors affect colonization, migration, and current population genetic structure. This study used 11 microsatellite loci to genotype 1,230 northern leopard frogs (*Rana pipiens*) from 41 wetlands (30 samples/wetland) across North Dakota. Genetic structure of the sampled frogs was evaluated using Bayesian and multivariate clustering methods. All analyses produced concordant results, identifying a major east–west split between two *R. pipiens* population clusters separated by the Missouri River. Substructuring within the two major identified population clusters was also found. Spatial principal component analysis (sPCA) and variance partitioning analysis identified distance, river basins, and the Missouri River as the most important landscape factors differentiating *R. pipiens* populations across the state. Bayesian reconstruction of coalescence times suggested the major east–west split occurred ~13–18 kya during a period of glacial retreat in the northern Great Plains and substructuring largely occurred ~5–11 kya during a period of extreme drought cycles. A range‐wide species distribution model (SDM) for *R. pipiens* was developed and applied to prehistoric climate conditions during the Last Glacial Maximum (21 kya) and the mid‐Holocene (6 kya) from the CCSM4 climate model to identify potential refugia. The SDM indicated potential refugia existed in South Dakota or further south in Nebraska. The ancestral populations of *R. pipiens* in North Dakota may have inhabited these refugia, but more sampling outside the state is needed to reconstruct the route of colonization. Using microsatellite genotype data, this study determined that colonization from glacial refugia, drought dynamics in the northern Great Plains, and major rivers acting as barriers to gene flow were the defining forces shaping the regional population structure of *R. pipiens* in North Dakota.

## 1 INTRODUCTION

Prehistoric climatic trends have played large roles in shaping the current biogeographic population structures of many North American species from a wide variety of lineages, including trees (Roberts & Hamann 2015), aquatic insects (Peterson, O'Grady, & Resh, 2017), fish (David & Wright, 2017; Tarpey et al., 2018), and mammals (Puckett, Etter, Johnson, & Eggert, 2015; Sim et al., 2016). As exothermic vertebrates dependent on both terrestrial and aquatic environments, amphibians are particularly susceptible to changes in the environment (Araújo et al., 2008; Mushet, Euliss, Chen, & Stockwell, 2013; Zeisset & Beebee, 2008). The signatures of past climate change are present in the genetic structure of many amphibian species (Ball et al., 2017; Lee‐Yaw, Irwin, & Green, 2008; Wielstra et al., 2013; Zeisset & Beebee, 2008; Zhou et al., 2017), and understanding how ancient climates affected current population structure can provide key insights into predicting how future climate change might impact amphibian populations.

Amphibians of the northern Great Plains provide an opportunity to evaluate prehistoric climate signatures on genetic variation. This region was partially glaciated and has been characterized by glacial retreat (Mickelson et al., 1983) followed by cycles of drought and wet periods throughout the Holocene (~11 kya – present; Valero‐Garcés et al., 1997; Xia, Haskell, Engstrom, & Ito, 1997). While the region southwest of the Missouri River was not glaciated, the region to the north and east was glaciated in the late Pleistocene until a rapid glacial retreat (~13 kya; Mickelson et al., 1983). This led to northward range expansions by a wide variety of species from southern refugia into newly deglaciated habitats (Masta, Laurent, & Routman, 2003; Wisely, Statham, & Fleischer, 2008; Yansa, 2006). The region remained cool and wet in the early Holocene following the glacial retreat (~11 kya–9 kya) before going through a prolonged extreme drought period (~9 kya–6 kya; Valero‐Garcés et al., 1997; Xia et al., 1997). Throughout the mid and late Holocene to the present, the climate of the northern Great Plains has been characterized by milder oscillations in precipitation, ending in a current wet period following drought during the Little Ice Age and the Medieval Warm Period (950–750 BP; Fritz, Engstrom, & Haskell, 1994; Xia et al., 1997).

The modern‐day northern Great Plains region encompasses a number of distinct ecoregions. The semi‐arid northwestern Great Plains is located to the south and west of the Missouri River and includes badlands, steppes, and shortgrass prairie with relatively low wetlands densities (Bryce et al., 1998; Euliss & Mushet, 2004). To the east of the Missouri River, there are the glaciated plains, which include the Prairie Pothole Region (PPR), an area with millions of depressional wetlands embedded primarily in shortgrass prairie that stretches from Saskatchewan to Nebraska and Iowa (Bryce et al., 1998; Tiner, 2003). Farther east, the shortgrass prairie transitions into tallgrass prairie and prairie pothole wetlands become less abundant on the Lake Agassiz Plain, an area formed after the draining of glacial Lake Agassiz ~8 kya (Bryce et al., 1998; Barber et al., 1999). Much of the native prairie and pothole wetlands in the Lake Agassiz Plain and the glaciated northern plains have been converted into small‐grain and row‐crop agriculture (~50% in North Dakota; Dahl, 1990), and are further threatened by continued agricultural expansion in the eastern part of the PPR and a potential shift to drier conditions due to climate change throughout much of the western part of the PPR (Carter Johnson et al., 2005, 2010).

The northern leopard frog (*Rana pipiens*) is a ranid frog species that is widely distributed throughout the temperate regions of North America (Hammerson et al. 2004). *Rana pipiens* use a variety of habitats throughout their life cycle. Breeding ponds are used by adults in the spring and by tadpoles throughout the summer (Dole, 1971). Adults and metamorph juveniles extensively use terrestrial habitats for foraging and migration between other habitats during the summer (Dole, 1971; Pope, Fahrig, & Merriam, 2000). *Rana pipiens* also require well‐oxygenated deep or flowing water habitats for overwintering hibernation (Cunjak, 1986; Emery, Berst, & Kodaira, 1972), which are particularly important for survival during the winter months on the northern Great Plains (Mushet 2010). Such habitats are rare during prolonged droughts, which limit the spatial distribution of northern leopard frog populations (Mushet 2010). *Rana pipiens* can migrate across relatively large distances among these different habitats, commonly moving 800 m, with reported ranges up to 5 km (Dole, 1971; Knutson, Herner‐Thogmartin, Thogmartin, Kapfer, & Nelson, 2018).

The genetic population structure and phylogeography of *R. pipiens* have been extensively studied and revised since the 1970s. Numerous described species were synonymized in the 1940s, but since the 1970s many species have been described based on morphology and/or genetics data (Hillis, 1988). Currently, *R. pipiens* has two distinct evolutionary lineages separated by the Mississippi River that were previously described as separate species (Cope, 1889; Hoffman & Blouin, 2004a; O'Donnell & Mock, 2012). Populations of *R. pipiens* east of the Mississippi River are typically more stable, have greater genetic diversity, and larger effective population sizes than populations west of the Mississippi River (Hoffman et al. 2004b; Philipsen et al. 2011, but see Mushet et al., 2013). Populations on the western edge of the range have become critically endangered and, in some cases, have already been extirpated (Corn & Fogleman, 1984; Rogers & Peacock, 2012). North Dakota lies in the transition zone between the more secure eastern populations and the imperiled western populations (NatureServe, 2017). Genetic diversity declines moving further west within the state of North Dakota, suggesting *R. pipiens* populations in the drier badlands and steppe habitats are at greater risk of extirpation than those in other parts of the state (Stockwell, Fisher, & McLean, 2016). Thus, it is useful to understand how spatial patterns in genetic variation have been shaped by post‐Pleistocene recolonization patterns and identify landscape features that constrain gene flow in order to determine how populations have responded to climate change.

This study applies population genetic tools on a finer scale than previous range‐wide studies to understand what regional landscape features and aspects of climate history have played important roles in how *R. pipiens* populations have been structured. This information will be useful for conservation efforts by delineating populations, identifying natural and anthropogenic barriers to gene flow, and assessing how climate‐related factors have influenced species distributions in the past. First, this study used microsatellite genotypes from *R. pipiens* (*N* = 1,230) from 41 sampling sites across North Dakota to determine whether *R. pipiens* in North Dakota were panmixic (H_01_) or clustered into genetically distinct populations (H_a1_). Then, spatial analyses were applied to the genetic data to determine whether isolation by distance alone (H_02_) or models that included modern‐day landscape features best described how the identified populations are spatially structured (H_a2_). Further, Bayesian coalescence models were used to evaluate likely divergence times for the defined genetic clusters. Finally, species distribution model for *R. pipiens* was developed to investigate how changes in climate and climate refugia may explain the evolutionary relationships between distinct populations of *R. pipiens*.

## 2 METHODS

### 2.1 Sample collection, DNA extraction, and microsatellite genotyping

**Figure 1:**
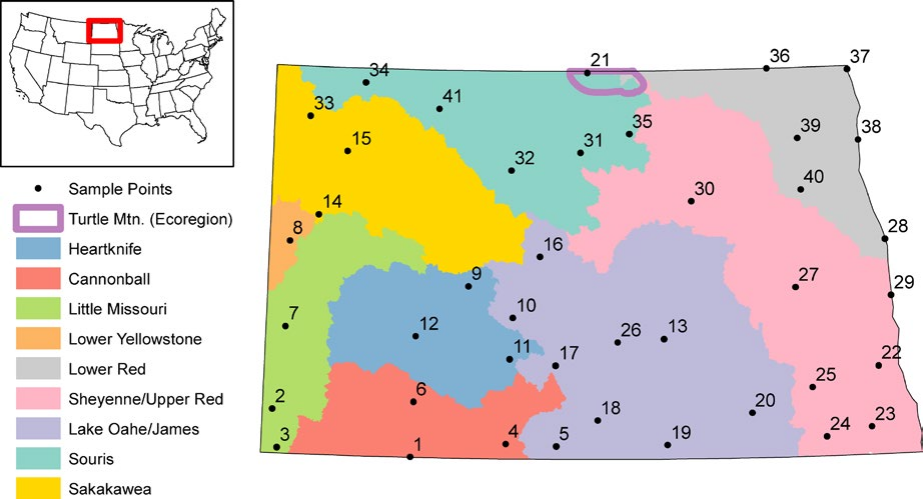
Sampling sites (*N* = 41) where genetic material was collected around the state of North Dakota. Colors represent the major river basins where *Rana pipiens* populations clustered together. The Turtle Mountain ecoregion is also outlined as the *R. pipiens* population that was sampled in that region clustered separately from the other sampled populations in the Souris basin

Forty‐one populations of *R. pipiens* were sampled throughout North Dakota (Figure 1). Potential sampling sites were selected a priori as permanent or semipermanent wetlands as classified by the US Fish & Wildlife Service National Wetland Inventory (USFWS, 2012; Fisher 2015). The distance between a sampling site and its nearest neighbor was at least 30 km and no greater than 85 km. At each site, neonate and adult specimens were captured by actively searching the wetland perimeter. Sampled specimens were spaced throughout the wetland perimeter to reduce the likelihood of sampling‐related individuals. *Rana pipiens* toe clips were collected from 30 individuals from each sampling site following NDSU IACUC protocol #A10047. Toe clips were stored in individually marked vials containing 95% ethanol.

Total genomic DNA was extracted and purified using DNeasy Blood and Tissue Kits (Qiagen, Hilden, Germany). Eleven microsatellite loci were amplified using primers developed by Hoffman, Arden, and Blouin (2003) for *R. pipiens *(Rpi 100, Rpi 101, Rpi 103, Rpi 104, Rpi 106, Rpi 107, Rpi 108), by Hoffman and Blouin (2004b) for the Oregon spotted frog (*Rana pretiosa*; RP197 and RP415), and by McKee, Lance, Jones, Hagen, and Glenn (2011) for the southern leopard frog (*Rana sphenocephala*; Rasp09 and Rasp20). Amplifications were conducted following the procedures published for each locus (Hoffman & Blouin, 2004b; Hoffman et al., 2003; McKee et al., 2011). Amplicons were visualized using an Applied Biosystems 3,130 automated sequencer. Resulting genotypes were visually assessed for automatic scoring accuracy, and 2% of samples were randomly rerun to ensure amplification consistency. The dataset was evaluated for genotyping errors and the presence of null alleles using MICRO‐CHECKER v. 2.2.3 (van Oosterhout, Hutchinson, Wills, & Shipley, 2004).

### 2.2 Analysis of genetic diversity and population structure

The genotype dataset was used to derive metrics of genetic diversity and population structure in North Dakota *R. pipiens*. Linkage disequilibrium (LD) and deviations in Hardy–Weinberg equilibrium (HWE) were assessed using GENEPOP’007 (Rousset, 2008). Expected heterozygosity and allelic richness for each sample site were calculated using the *adegenet *package (Jombart, 2008) in R v.3.4.1 (R Core Team 2017). Nei's genetic distance (G_ST_) was calculated between each sampling site and was used to construct an Unweighted Pair Group Method with Arithmetic Mean (UPGMA) tree using the *phangorn* package (Schliep, 2011).

Population structure was examined using two Bayesian clustering algorithms, STRUCTURE 2.3 (Pritchard, Stephens, & Donnelly, 2000) and BAPS 3.2 (Corander, Sirén, & Arjas, 2008), and a multivariate method using K‐means clustering following principal component analysis (PCA) of the microsatellite dataset in R. These three methods represent three statistically distinct approaches to describe population structure, allowing consistent patterns of structure to be distinguished from statistical artifacts of the clustering method. STRUCTURE and BAPS rely on different Bayesian clustering algorithms, the main difference being that BAPS has been optimized for incorporating spatial data into the clustering algorithm as a model parameter (Corander et al., 2008). The multivariate method does not rely on Bayesian inference, but instead uses dimensional reduction through PCA and a successive K‐means clustering to determine numbers of populations and classify individuals into populations (Corander et al., 2008).

STRUCTURE analysis consisted of an admixture model with correlated allele frequencies for each potential number of clusters (*K*). Each analysis consisted of 200,000 simulations after an initial burn‐in of 20,000 simulations. The analysis was run for *K* values ranging from 1 to 41 possible clusters with 10 independent runs each. The Δ*K* method (Evanno, Regnaut, & Goudet, 2005) was used to identify the best‐supported *K* value, which was determined based on the *K* value with the greatest ratio of change in the posterior probabilities of two sequential *K* values. If inconsistent results in *K* values were found compared to BAPS, additional nested STRUCTURE analyses were performed individually within each *K* group identified by the previous STRUCTURE analysis (Breton, Pinatel, Médail, Bonhomme, & Bervillé, 2008; Pereira‐Lorenzo et al., 2010). These additional STRUCTURE analyses allowed for identification of potential substructuring that may have been missed.

BAPS analyses were initially performed by both “clustering of individuals” and “spatially clustering of individuals” models within the population mixture analysis. These analyses were performed using *K*_max_ values ranging from 1 to 41 with 10,000 iterations per run to estimate the admixture coefficients of each sample. Ten replicates were performed for each value of *K*_max_ to investigate the consistency of results for all values of *K*.

The *adegenet* package in R was used to perform multivariate analysis of population structure (Jombart, 2008). Multivariate analysis of the microsatellite data first required the reduction of dimensions using PCA, which was performed by the dudi.pca function from the *ade4* R package (Chessel, Dufour, & Thioulouse, 2004). The first 100 principal components (PCs) explaining >95% of the variation in the microsatellite dataset were retained to use in K‐means clustering. Clustering for each *K* value was evaluated using BIC (Bayesian information criterion), and the *K* value with the lowest BIC value was selected for further evaluation. Sample sites were classified into populations based on which cluster the majority (>50%) of individuals from the sample site were assigned. For sample sites where no cluster represented a majority of individuals, the top two clusters within that sample site were combined into one cluster and this was applied to all samples across the dataset. This process was repeated until all sample sites contained a majority of individuals from one cluster. The identified population clusters were used as predefined groups for discriminant analysis of principal components (DAPC; Jombart, Devillard, & Balloux, 2010) using the *adegenet* package in R (Jombart, 2008). This method reduces the dimensionality of the genetic variation between groups using PCA and then uses the PCs produced from this analysis in a linear discriminant analysis (LDA) to create discriminant functions representing a linear combination of correlated alleles that describe the greatest amount of variation in the genetic dataset. The optim.a.score function was used to determine the optimal number of PCs to retain to best describe the population structure without overfitting the discriminant functions. Population assignment probabilities were calculated for the optimized DAPC model, the hierarchical STRUCTURE model, and the BAPS model to assess how clearly populations were discriminated using each method.

### 2.3 Landscape genetic analysis

Pairwise *F*_ST_ (Nei, 1973) values were calculated for each pair of sampling sites as a measure of genetic distance between the frogs from each sampling site. Linear geographic distance between each pair of sampling sites was also calculated. A Mantel test including the linearized *F*_ST_ values [*F*_ST_/(1‐*F*_ST_)] and geographic distances was performed to find evidence for isolation by distance in the *R. pipiens* sampling sites across the state using the mantel function from the *vegan* package in R (Oksanen et al. 2017). A partial Mantel test was also performed to control for the variation associated with geographic distance and test the effect of the Missouri River as a barrier. Sample sites were coded into a barrier matrix with a binomial variable representing sites on the same side (0) or opposite sides (1) of the Missouri River.

To test for global and local population spatial structure, a spatial principal component analysis (sPCA) was performed on the *R. pipiens* genetic dataset (Jombart, Devillard, Dufour, & Pontier, 2008). The sPCA works by maximizing the product of variance in allele frequencies and spatial autocorrelation (Moran's I) to find groups of alleles that are correlated with each other through space. An inverse square distances connection network was established to characterize the spatial relationships between each sampling site in the sPCA. The abilities of the eigenvalues produced by the sPCA to explain spatial population structure were assessed using global and local Monte Carlo tests (Jombart et al., 2008). Three principal components with the largest positive eigenvalues were retained for further analysis.

Landscape features affecting ecological suitability and the ability of *R. pipiens* to move between populations were included in a redundancy analysis (RDA; Legendre & Legendre, 2012) using the three retained sPCA principal components as a measure of genetic variation of the populations across the state. Landscape factor variables included in the RDA model were land use type, the Missouri River as a barrier, and the 6‐digit hydrologic unit code (HUC‐6) basin within which the sampling site was located. The land use type variable was created by drawing a 15 km buffer around each sampling site in ArcMap v10.5 (ESRI). This distance represents an area where high levels of gene flow (≥1 migrant/generation) would be expected between ponds within the buffer based on the distance *R. pipiens* can disperse within one generation (Dole, 1971; Knutson et al., 2018). Land use types from the National Land Cover Database (Homer et al., 2015) were reclassified into six classes: open water, urban/developed land, forest, scrubland/grassland, agriculture, and wetlands. The area of these classes within each 15 km buffer was calculated and converted into proportions to use as the land use variable in the RDA model. The Missouri River variable was created by classifying sampling sites as being east or west of the Missouri River. Finally, the basin variable was created by classifying sampling sites based on their HUC‐6 location.

A full RDA model including latitude and longitude positions of each sampling site along with all landscape features was run to determine whether any landscape variables explained a significant amount of variation in the sPCA axes. A partial RDA model conditioned on the latitude and longitude of sampling sites was run with all of the landscape feature variables to partial out variation in the sPCA axes due to isolation by distance to determine whether the selected landscape variables explained a significant amount of the remaining variation. Additional partial RDA models were run with each of the landscape factor as a single constrained variable conditioned on the other landscape factors, partitioning the variance in sPCA scores explained by each landscape factor on its own. A permutated ANOVA (PERMANOVA; Legendre, Oksanen, & Braak, 2011) was performed on each partial RDA model result with 999 permutations to determine whether the single unconditioned landscape factor in the partial RDA model explained a significant amount of the variance in the sPCA axes scores.

### 2.4 Historical population coalescence and paleoclimate modeling

**Figure 2:**
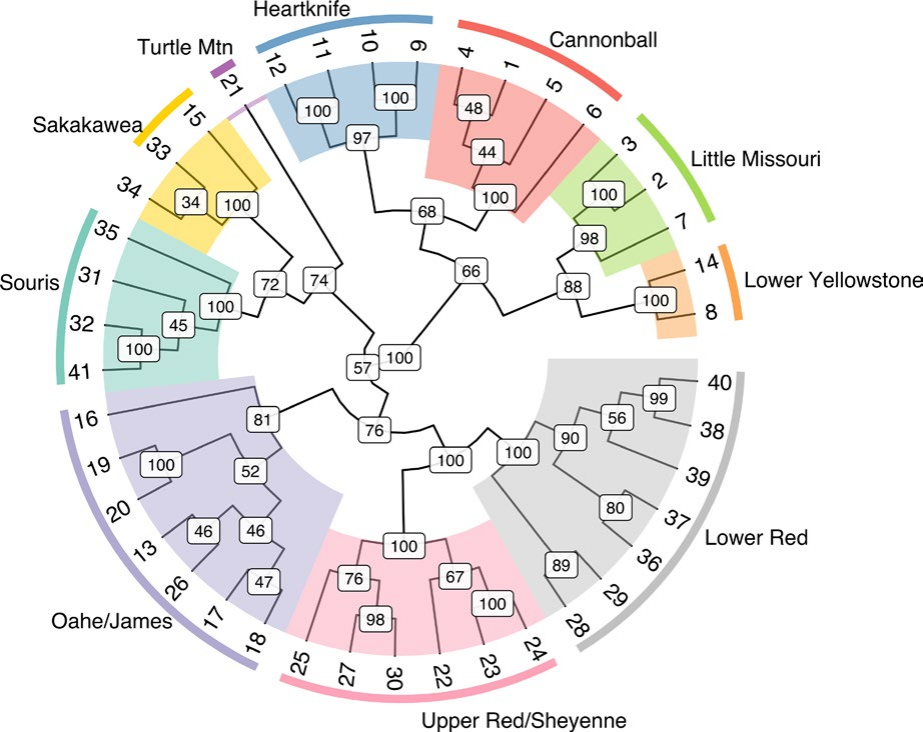
Phylogenetic UPGMA tree of 41 sampled populations of *Rana pipiens*. Population clusters by basin are outlined by color matched to the basins in Figure 1

Population structure is strongly influenced by population demographic history, so the times of coalescence between the identified populations were estimated. DIYABC v2.0.4 (Cornuet et al., 2008), an approximate Bayesian computation (ABC) method (Beaumont 2008; Bertorelle, Benazzo, & Mona, 2010; Csilléry, Blum, Gaggiotti, & François, 2010), was used to estimate the coalescence times of the populations using the topology of the UPGMA tree (Figure 2). One million multilocus datasets based on two different sets of summary statistics were simulated. The first model used a set of summary statistics based on Beaumont (2008) and included the mean number of alleles, heterozygosity, and allele size variance. The second model was based on the set of summary statistics including mean number of alleles, heterozygosity, and Garza–Williamson's M, as well as two sample statistics including *F*_ST_ and individual likelihood assignment (the likelihood an individual from population *i* would be assigned to population *j*). A stepwise mutation model with a average mutation rate of 10^−3^–10^−4^ was used to describe mutation dynamics across the whole set of microsatellite markers, while individual marker mutation rates ranged from 10^−2^ to 10^−5^, representing a range of mutation rates commonly seen in vertebrates, including amphibians (Bulut et al., 2009; Guillemaud, Beaumont, Ciosi, Corneut, & Estoup, 2010; Storz & Beaumont, 2002). Uniform priors were set for population size (1–20,000 individuals) and time of coalescence (1–20,000 generations). *Rana pipiens* generation time is approximately one year, putting the maximum prior for coalescence at the Last Glacial Maximum when the area of North Dakota east of the Missouri River was glaciated and could not support *R. pipiens* populations.

Population divergence for many species in North America has been driven by climatic shifts and glacial retreats in the past 20,000 years. A species distribution model was used to explore how climate change has affected the amount of suitable habitat for *R. pipiens*. To create a species distribution model, observation records of *R. pipiens* were downloaded from VertNet (Florida Museum of Natural History et al. 2017). Observations outside the United State and Canada and observations from the states of Florida, Alabama, Georgia, Mississippi, Louisiana, and Texas were removed from the dataset as they were likely erroneous observations or were observations of southern leopard frog (*Rana sphenocephalus*) before it was classified as a separate species. The remaining observations were also screened for duplicate records from the same location and records from fossils or captive observations, resulting in *N* = 801 final observations to use in habitat suitability modeling (Supporting Information Figure S1).

Four habitat suitability modeling methods and bioclimatic data from the WorldClim database (Fick & Hijman 2017) were used to generate the species distribution model. The first modeling method used was a binomial generalized linear model. To create a dataset for this model to analyze, 800 background points were randomly distributed throughout the continental United States, Canada, and Mexico. The values of 19 bioclimatic variables were extracted from the locations of each observation and background point. The binomial logistic regression model classified each point as an observation or a background point for the dependent variable and used the bioclimatic variables as independent variables. Variance inflation factors (VIFs) were calculated for the bioclimatic variables since it is likely that some climatic variables would be highly correlated, and the variable with the highest VIF was removed until all VIF values were <10. The binomial logistic regression model was then run with all possible combinations of the remaining bioclimatic variables and assessed with BIC. Models with BIC values <2 were averaged together to produce a final binomial logistic regression model. BIOCLIM (Booth, Nix, Busby, & Hutchinson, 2014), Mahalanobis distance (Mahalanobis, 1936), and Maxent (Philips et al. 2006) models were implemented using the dismo package in R (Hijmans, Phillips, Leathwick, & Elith, 2017) using only the observed locations. No background points are necessary to fit these types of models. BIOCLIM and Mahalanobis distance models were implemented with the same bioclimatic variables the binomial logistic regression model retained after stepwise VIF variable selection. Maxent models were run using a suite of bioclimatic variables that explained at least 5% of the variation in a Maxent model that included all 19 bioclimatic variables. R code and specific conditions for each model are included in Supporting Information Figure S1.

Model validation for all models was carried out using a k‐fold sampling scheme. Points were randomly assigned a *K* value of one through four, and four separate model optimization trials were conducted. In each trial, a subset of points assigned one of the *K* values was left out to validate the model after it was fit, and a subset of points with the three other *K* values was used as a training dataset to build the model. This process was iterated four times for each modeling method, each time leaving out a different subset of the presence and background points. The area under the curve (AUC) for each fitted model was calculated using the *K* group of validation points left out during model fitting and was used to create a weighted average of all model results [*w* = (AUC − 0.5)^2^]. Models predictions of suitability were rescaled to the same scale and averaged together using the weighted average to produce the final maps of habitat suitability. The averaged models were used to estimate habitat suitability during the present day, Last Glacial Maximum (~21 kya), and the mid‐Holocene (~6 kya) using publicly available bioclimate variable layers produced by the CCSM4 historical model scenarios (Hijmans, Camseron, Parra, Jones, & Jarvis, 2005) downloaded using the *sdmpredictors* package in R (Bosch, 2017).

## 3 RESULTS

### 3.1 Genetic diversity and population structure

No null alleles, significant deviations from Hardy–Weinberg equilibrium, or evidence of linkage disequilibrium was observed for any of the 11 loci. Allelic richness for each locus varied from 3 to 28 with an average of 17.8 alleles per locus (Table 1). The overall observed heterozygosity (H_o_) across all sites and all loci was 0.768, and overall expected heterozygosity (*H_e_*) was 0.788. Observed heterozygosity within each sampling site ranged from 0.661 to 0.882 (Table 2). The inbreeding coefficient (*F_IS_*) within sample sites varied from −0.0706 to 0.1146 (overall = 0.0259).

**Table 1:** Allelic richness and heterozygosities of microsatellite loci genotyped for each population of *Rana pipiens* sampled from 41 sites across North Dakota

Locus	Allelic richness	Expected heterozygosity (*H*_e_)	Observed heterozygosity (*H*_0_)
Rpi 100	17	0.90	0.80
Rpi 101	13	0.87	0.84
Rpi 103	23	0.93	0.76
Rpi 104	3	0.55	0.48
Rpi 106	13	0.89	0.76
Rpi 107	15	0.82	0.80
Rpi 108	28	0.91	0.82
RP197	20	0.91	0.85
RP415	24	0.93	0.72
Rasp09	17	0.90	0.83
Rasp20	23	0.93	0.79

**Table 2:** Measures of genetic diversity and population cluster assignment of the majority of individuals from each sampling site

Sample site ID	Observed heterozygosity	Expected heterozygosity	Inbreeding coefficient (*F*_IS_)	Population assignment
1	0.678	0.711	0.0483	Cannonball
2	0.661	0.718	0.0726	Little Missouri
3	0.664	0.707	0.0599	Little Missouri
4	0.709	0.731	0.0292	Cannonball
5	0.718	0.698	−0.0355	Cannonball
6	0.693	0.696	−0.0014	Cannonball
7	0.691	0.744	0.0688	Little Missouri
8	0.773	0.776	−0.0010	Lower Yellowstone
9	0.794	0.757	−0.0517	Heartknife
10	0.800	0.766	−0.0416	Heartknife
11	0.776	0.753	−0.0347	Heartknife
12	0.758	0.771	0.0149	Heartknife
13	0.788	0.804	0.0224	Oahe/James
14	0.824	0.777	−0.0651	Lower Yellowstone
15	0.757	0.797	0.0482	Sakakawea
16	0.794	0.823	0.0365	Oahe/James
17	0.733	0.806	0.0868	Oahe/James
18	0.715	0.794	0.1000	Oahe/James
19	0.730	0.827	0.1146	Oahe/James
20	0.739	0.818	0.0935	Oahe/James
21	0.766	0.717	−0.0730	Turtle Mountain
22	0.812	0.833	0.0264	Upper Red/Sheyenne
23	0.785	0.850	0.0809	Upper Red/Sheyenne
24	0.824	0.854	0.0356	Upper Red/Sheyenne
25	0.766	0.820	0.0673	Upper Red/Sheyenne
26	0.776	0.811	0.0393	Oahe/James
27	0.739	0.833	0.1123	Upper Red/Sheyenne
28	0.803	0.820	0.0195	Lower Red
29	0.809	0.816	0.0071	Lower Red
30	0.739	0.824	0.1047	Upper Red/Sheyenne
31	0.727	0.799	0.0870	Souris
32	0.755	0.806	0.0619	Souris
33	0.763	0.761	−0.0024	Sakakawea
34	0.792	0.779	−0.0164	Sakakawea
35	0.752	0.812	0.0715	Souris
36	0.855	0.822	−0.0432	Lower Red
37	0.882	0.825	−0.0706	Lower Red
38	0.875	0.824	−0.0654	Lower Red
39	0.860	0.828	−0.0394	Lower Red
40	0.857	0.817	−0.0502	Lower Red
41	0.743	0.790	0.0608	Souris
Overall	0.768	0.788	0.0259	—

**Figure 3:**
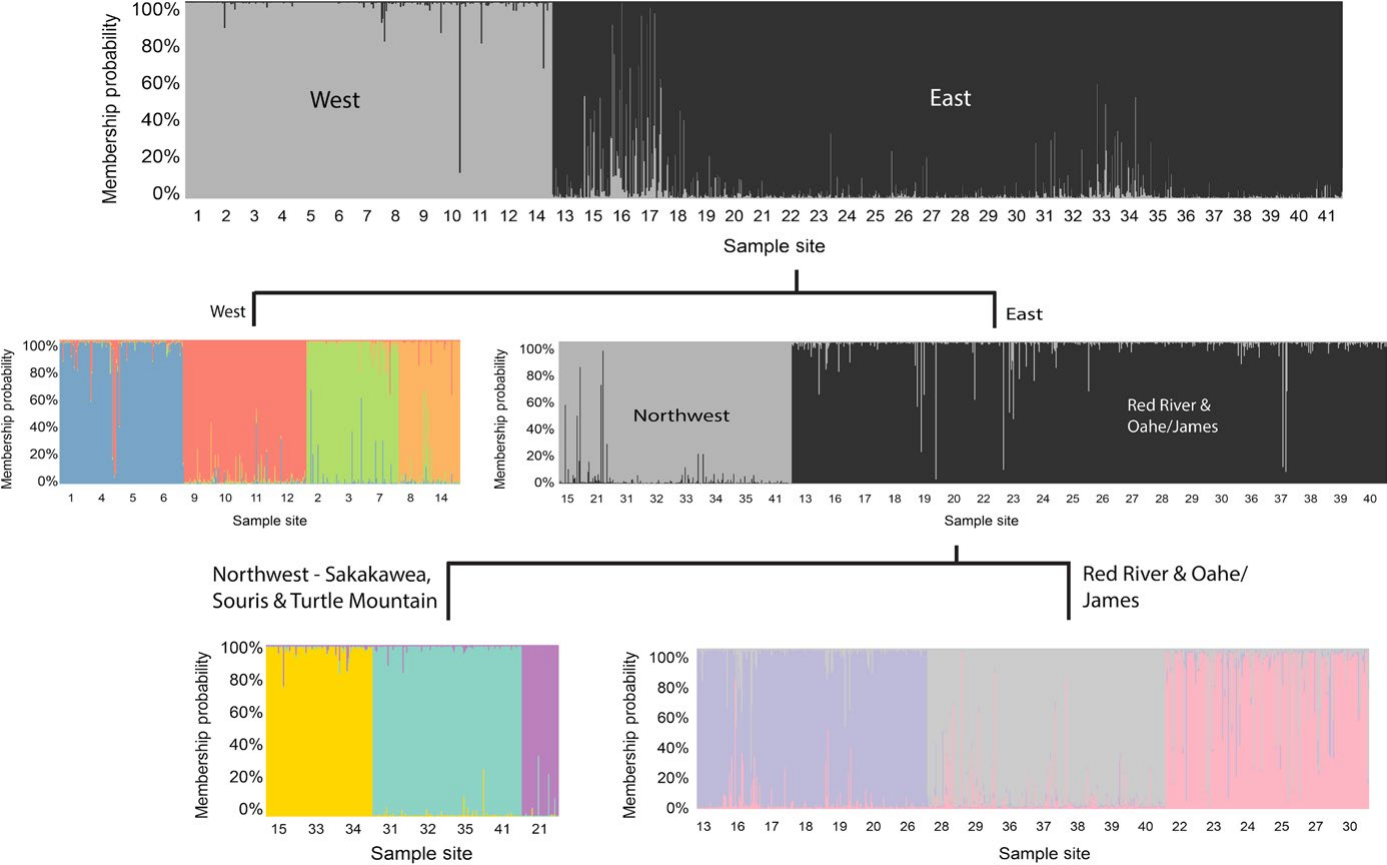
Results of hierarchical STRUCTURE population clustering. The initial STRUCTURE run split the populations of *Rana pipiens* in North Dakota into two main clusters separated by the Missouri River; Populations 1–12 and 14 were to the southwest, whereas all remaining populations were northeast of the Missouri. The populations west of the Missouri River broke into four clusters when analyzed separately. The populations east of the Missouri River clustered into two large groups, one in the northwestern part of the state and one in the southern and eastern parts of the state. Each of these two groups further clustered into three clusters largely separated by basin

**Figure 4:**
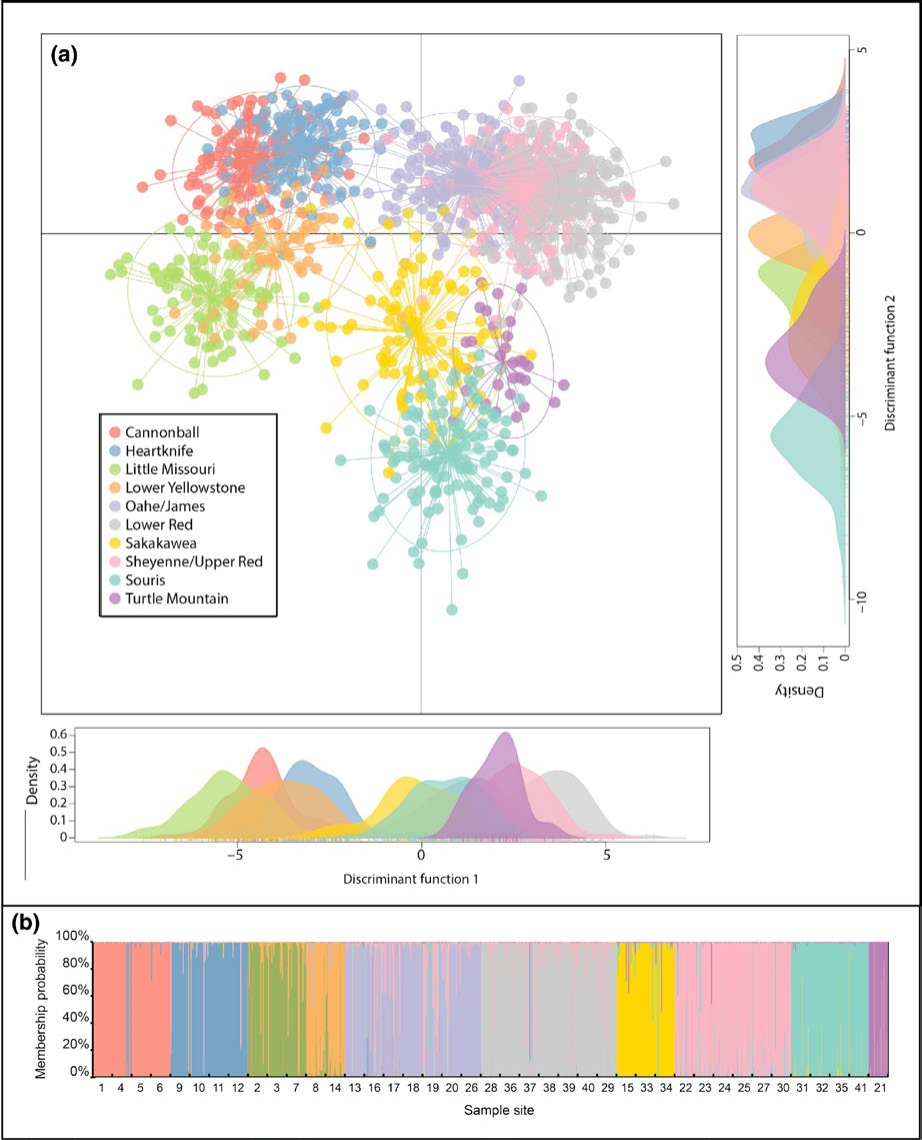
Results from DAPC population clustering analysis. (a) The first two discriminant functions explained 37.6% and 21.5% of the genetic variation in *Rana pipiens* from the sampled sites. Each node represents the genotype of an individual frog connected to a centroid of the cluster the frog was assigned to based on K‐means clustering of the DAPC scores. (b) DAPC determined the sampled individuals were optimally clustered into ten groups, with 99.6% of individuals being assigned to one of these clusters with Q > 0.5.

Populations were strongly structured (mean pairwise *F*_ST_ = 0.0930), and all population structure analyses produced concordant results. The UPGMA tree (Figure 2) provided clear evidence of two primary branches that corresponded to population clusters northeast and southwest of the Missouri River. The tree also shows that major branches generally align with HUC‐6‐level basins. These structural patterns were corroborated with the STRUCTURE analysis results, which produced an initial K of 2, splitting populations northeast and southwest of the Missouri River. Hierarchical STRUCTURE analysis within the two main populations produced *K* = 4 subpopulations southwest of the Missouri River and *K* = 6 subpopulations northeast of the Missouri River that corresponded with the major branches on the UPGMA tree (Figure 3). BAPS analysis results were consistent with the hierarchical STRUCTURE analysis, producing a total of *K* = 10 clusters. Principal component analysis (PCA) and *K*‐means clustering determined *K* = 16 clusters to have the lowest BIC. Further analysis of the *K* = 16 clusters revealed some of the clusters did not constitute a majority within any one site. Once the clusters were combined into majorities within each site, the PCA results matched the STRUCTURE and BAPS analyses with the same 10 major clusters. Discriminant analysis of the principal components (DAPC) also suggested hierarchical structure. The first discriminant function supported the east–west divide around the Missouri River found by the other analyses and explained 37.6% of the total genetic variance (Figure 4a). More fine‐scaled structure within the main populations detected by all three clustering methods produced clusters that were largely associated with HUC‐6‐level basins (Figure 4b). Four of these clusters are associated with the Lower Yellowstone, Little Missouri, and split the Cannonball–Heartknife basin southwest of the Missouri River, while the other six clusters northeast of the Missouri River were associated with the Souris, Sakakawea, Oahe/James, Upper Red/Sheyenne, and Lower Red River basins. There was one sample site from the Turtle Mountain region on the northern border of the state that clustered alone despite falling in the Souris basin.

Membership assignment of individuals to the ten identified populations was consistent among each structuring analysis method. Nearly all of the individuals were assigned to a population (Q > 0.5) in the hierarchical STRUCTURE, BAPS, and DAPC (99.3%, 99.1%, and 99.6% of individuals, respectively). Hierarchical STRUCTURE, BAPS, and DAPC assigned individuals to the same population cluster with membership probability (Q > 0.5) 89.0% of the time. The two Bayesian clustering methods, STRUCTURE and BAPS, agreed on population assignment of individuals 95.7% of the time. DAPC assigned individuals to the same populations as STRUCTURE and BAPS 90.6% and 90.7% of the time, respectively. Because results were so similar, further analysis of population structure will be reported using results from DAPC only.

Average membership assignment probability to each population cluster ranged from 0.967 to 0.817. Individuals from the Upper Red/Sheyenne and the Oahe/James populations had the lowest average probabilities of assignment (0.817 and 0.852, respectively). The majority of individuals from the Upper Red/Sheyenne population assigned to a different population were either assigned to the Lower Red (12.2% of Upper Red/Sheyenne individuals) or the Oahe/James population (3.8%). Likewise, most of the discordant individuals from the Oahe/James came from the Upper Red/Sheyenne (5.2% of Oahe/James individuals) or the Lower Red population (4.3%). The high rate of cross‐assignment of individuals within these bordering river basins suggests there may be admixture between these populations. There were two individuals from sample site 17 on the east side of the Missouri that had a genotype with significant assignment probabilities (>0.4) to populations to the west of the Missouri River, suggesting there may be some limited dispersal across the Missouri River between the Oahe/James and Cannonball–Heartknife populations.

### 3.2 Landscape effects on population structure

**Figure 5:**
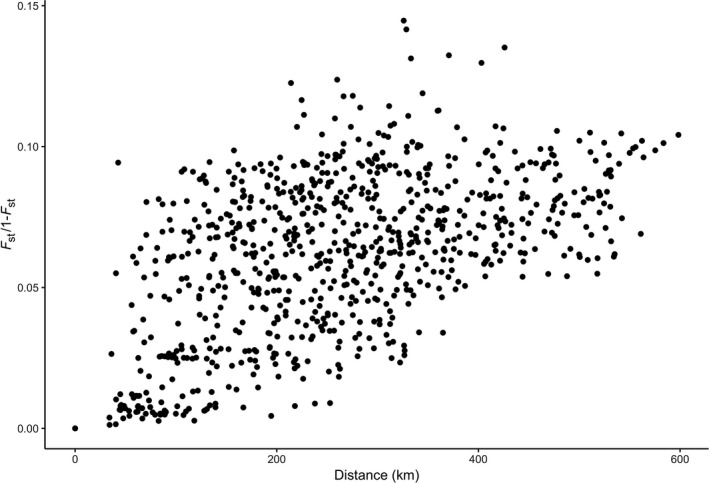
Correlation between the geographic distance and linearized pairwise *F*_ST_ for the 41 sampled populations of *Rana pipiens* in North Dakota. There is a strong signature of isolation by distance with *F*_ST_ generally increasing with geographic distance

The Mantel test indicated there was significant isolation by distance (IBD) among sample sites (*r* = 0.515, *p* < 0.001). Geographic distance was positively correlated with genetic distance (Figure 5). The partial Mantel test corroborated the east–west divide around the Missouri River seen in the population structuring analyses. There was a significant isolation‐by‐barrier effect between sites on opposite sides of the Missouri River (*r* = 0.582, *p* < 0.001).

**Figure 6:**
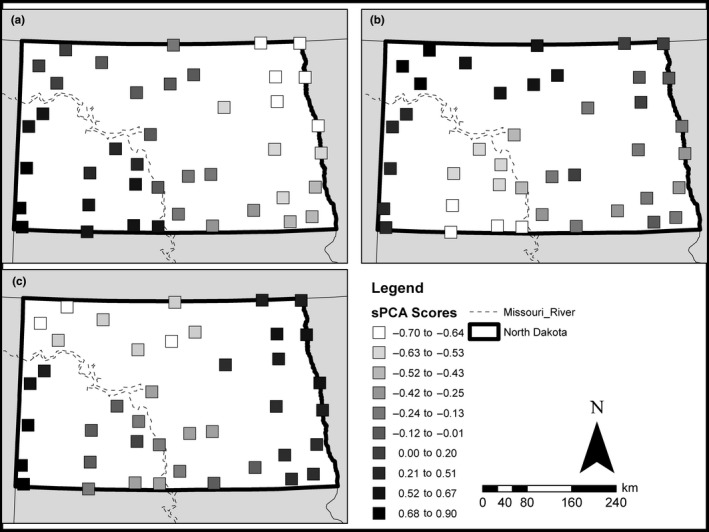
Results from sPCA landscape analysis showing sPCA scores for the most important sPCA axes describing spatial genetic variation in the 41 sample sites. Scores from the first sPCA axis (a) explained 47.3% of the spatial genetic structure of *Rana pipiens* in the state of North Dakota indicating a divide between sampled sites on opposite sides of the Missouri River. The second (b) and third (c) sPCA axes explained 23.0% and 14.4% of the genetic structure, respectively, and both depict how the major east and west populations were substructured, with the greatest differentiation in sPCA scores seen between the westernmost populations in the southwest (Lower Yellowstone and Little Missouri basins) and the populations to the east of the Badlands (Heartknife and Cannonball basins), and differentiation seen between populations in the northwest (Sakakawea and Souris basins) from populations in the Red River basin, while populations in the Oahe and James basins show more intermediate genotypes

Spatial PCA found strong global structuring in the genetic variation across all sampling sites (global Monte Carlo test; *r*_obs_=0.146, *p* < 0.001), and there was no evidence of local structuring within sampling sites (local Monte Carlo test; *r*_obs_=0.036; *p* = 906). The top three sPCA axes that were retained explained 84.6% of the spatial genetic structure. The first sPCA axis explained 47.3% of the spatial genetic structure and indicated a stark split between populations on opposite sides of the Missouri River (Figure 6a). The second sPCA axis explained 23.0% of spatial variation and showed similarities between the Souris and Sakakawea population cluster with the Little Missouri and Lower Yellowstone cluster. The second sPCA axis also shows a stark division between the Cannonball–Heartknife populations and the other populations west of the Missouri River along with a gradual change from the Cannonball–Heartknife populations to the populations in the Red River Valley (Figure 6b). The third sPCA axis explained 14.4% of the spatial variation and differentiated the Souris and Sakakawea population clusters from the Lower Yellowstone and Little Missouri populations (Figure 6c).

The full RDA model explained 97.6% of the variation in the first three sPCA axes (*pseudo‐F* = 48.7, *p* = 0.001). The partial RDA conditioned on the geographic coordinates of sites found a significant effect of landscape variables after removing the effects of isolation by distance (*pseudo‐F* = 20.7, *p* = 0.001). The IBD effect accounted for 60.8% of the variance in the sPCA axes, 36.8% was explained by the landscape factors, and 2.4% was unexplained. Partial RDA models focusing on individual landscape variables found two variables that explained a significant amount of variation in the sPCA axes (Table 3). HUC‐6 basin explained 21.0% of the variance (*pseudo‐F* = 21.0, *p* = 0.001), and the Missouri River explained 4.0% of the variance (*pseudo‐F* = 35.7, *p* = 0.001). None of the land use types explained a significant amount of variance.

**Table 3:** Results of partial RDA models and permuted ANOVA significance tests for individual landscape variables explaining the variation in spatial PCA axes

Variable	% Variance explained	Pseudo‐*F*	*p* value
Latitude + Longitude (isolation by distance)	60.8	—	—
Open Water (proportion within 5 km)	0.1	0.459	0.665
Urban Land Use (proportion within 5 km)	0.0	0.432	0.708
Forest (proportion within 5 km)	0.1	0.459	0.663
Scrub/Grassland (proportion within 5 km)	0.1	0.463	0.659
Agricultural Land Use (proportion within 5 km)	0.1	0.469	0.653
Wetlands (proportion within 5 km)	0.1	0.638	0.538
Missouri River barrier	4.0	35.685	0.001*
HUC−6 Basins	21.0	21.009	0.001*

^*^Indicates statistical significance of the PERMANOVA result (*p* < 0.05).

### 3.3 Population coalescence

**Figure 7:**
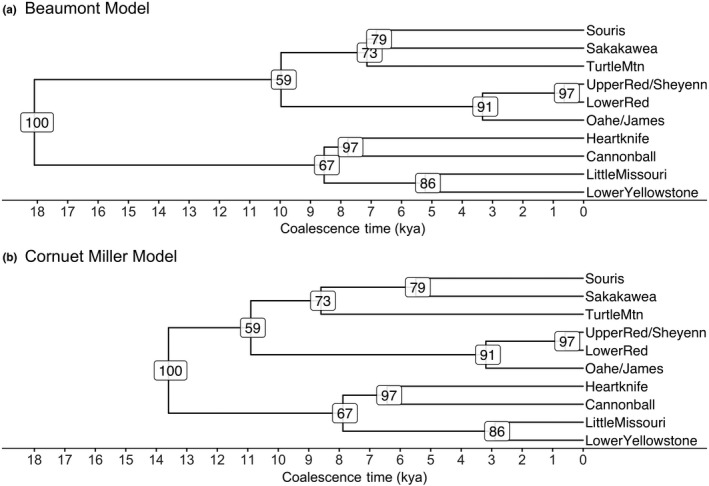
Coalescence trees of the ten major clusters of *Rana pipiens* in North Dakota based on the Beaumont (a) and Cornuet–Miller (b) models. Both models show the major split between the populations east and west of the Missouri River occurred during the late Pleistocene (18–13 kya), while most of the subdivision within these major populations occurring during the dry period of the Holocene (11–7 kya)

DIYABC indicated that median coalescence times among all 10 population clusters varied from 638 to 18,100 generations for the Beaumont model (Figure 7a; Table 4) and 588 to 13,600 generations for the Cornuet–Miller model (Figure 7b; Table 4). The median coalescence time for the southwestern and northeastern clusters separated by the Missouri River was 13,600 generations and 18,100 generations for the Cornuet–Miller and Beaumont models, respectively (Figure 7; Table 4). For populations east of the Missouri River, median coalescence times for the Cornuet–Miller model varied from 588 to 10,900 generations or 638 to 9,970 using the Beaumont model. For populations west of the Missouri, the Cornuet–Miller model estimated median coalescence times varying from 2,980 to 6,430 generations, and the Beaumont model estimated coalescent times from 5,220 to 8,550 generations. *Rana pipiens* have a generation time of ~1 year. This puts the time of divergence between the east and west populations in the late Pleistocene after the Last Glacial Maximum, and most of the divergence times between the subpopulations occurred during the early to mid‐Holocene.

**Table 4:** Time of divergence estimates for northern leopard frog (*Rana pipiens*) populations in North Dakota

Time	Converged units	Beaumont model median convergence time in generations (95% CI)	Cornuet–Miller model median convergence time in generations (95% CI)
1	Lower Red (E) and Upper Red/Sheyenne (E)	638 (129, 5,770)	588 (128, 4,630)
2	Little Missouri (W) and Lower Yellowstone (W)	5,220 (1,470, 13,400)	2,890 (730, 8,850)
3	Heartknife (W) and Cannonball (W)	7,770 (2,320, 14,200)	7,880 (2,390, 13,700)
4	Red River/Sheyenne Basins (E) and James/Oahe (E)	3,230 (685, 14,000)	3,190 (780, 13,200)
5	Sakakawea (E) and Souris (E)	7,140 (2,220, 14,600)	5,490 (1,650, 12,600)
6	Missouri/Yellowstone (W) and Cannonball/Heartknife (W)	8,550 (3,470, 16,400)	6,430 (2,620, 14,200)
7	Sakakawea/Souris (E) and Turtle Mountains (E)	6,830 (2,000, 14,400)	8,600 (3,200, 15,700)
8	Northeast (E) and Red River/James/Oahe (E)	9,970 (3,620, 19,000)	10,900 (4,290, 19,100)
9	All Western Populations (W) and all Eastern Populations (E)	18,100 (13,100, 19,900)	13,600 (6,390, 19,600)

Note

E, population located east of the Missouri River; W, population located west of the Missouri River.

### 3.4 Species distribution modeling and paleoclimate projections

Sixteen models were included in the final averaged model (Table 5). For the binomial logistic regression models, there was a maximum of two models with ΔBIC<2, which were then averaged together. All of the averaged binomial logistic regression models included the same six bioclimatic variables, three related to temperature and three related to precipitation. Two precipitation variables, precipitation during the driest month and precipitation seasonality, were present in all of the best performing models and were most important in defining the suitable range of *R. pipiens*. Precipitation during the driest month was positively associated with *R. pipiens* occurrence, which also had a consistent negative relationship between with precipitation seasonality, suggesting *R. pipiens* depended on moderate to high levels of precipitation throughout the year.

**Table 5:** Binomial logistic regression (GLM), BIOCLIM, Mahalanobis distance (Mahal), and Maxent models included in the final averaged species distribution model with variables included (x), the AUC score, and model weight for each model. GLM models have a (+) or (−) instead of an (x) to represent the relationship a variable has with the probability of *R. pipiens* presence

Model	Bio1—Annual Mean Temperature	Bio2—Mean Diurnal Range	Bio3—Isothermality	Bio4—Temperature Seasonality	Bio5—Maximum Temperature of Warmest Month	Bio7—Temperature Annual Range	Bio8—Mean Temperature of Wettest Quarter	Bio9—Mean Temperature of Driest Quarter	Bio10—Mean Temperature of Warmest Quarter	Bio11—Mean Temperature of Coldest Quarter	Bio13—Precipitation of Wettest Month	Bio14—Precipitation of Driest Month	Bio15—Precipitation Seasonality	Bio17—Precipitation of Driest Quarter	Bio18—Precipitation of Warmest Quarter	AUC	Model Weight (AUC−0.5)^2^
GLM1					+		−	−			−	+	−			0.782	0.080
GLM2					+		−	−			−	+	−			0.792	0.085
GLM3					+		−	−			−	+	−			0.792	0.085
GLM4					+		−	−			−	+	−			0.770	0.073
Bioclim1		x		x	x		x	x			x	x	x		x	0.852	0.124
Bioclim2		x		x	x		x	x			x	x	x		x	0.834	0.111
Bioclim3		x		x	x		x	x			x	x	x		x	0.854	0.125
Bioclim4		x		x	x		x	x			x	x	x		x	0.844	0.118
Mahal1		x		x	x		x	x			x	x	x		x	0.901	0.161
Mahal2		x		x	x		x	x			x	x	x		x	0.902	0.162
Mahal3		x		x	x		x	x			x	x	x		x	0.894	0.155
Mahal4		x		x	x		x	x			x	x	x		x	0.889	0.152
Maxent1	x	x	x	x		x			x	x		x	x	x		0.897	0.158
Maxent2	x	x	x	x		x			x	x		x	x	x		0.902	0.161
Maxent3	x	x	x	x		x			x	x		x	x	x		0.895	0.156
Maxent4	x	x	x	x		x			x	x		x	x	x		0.898	0.158

**Figure 8:**
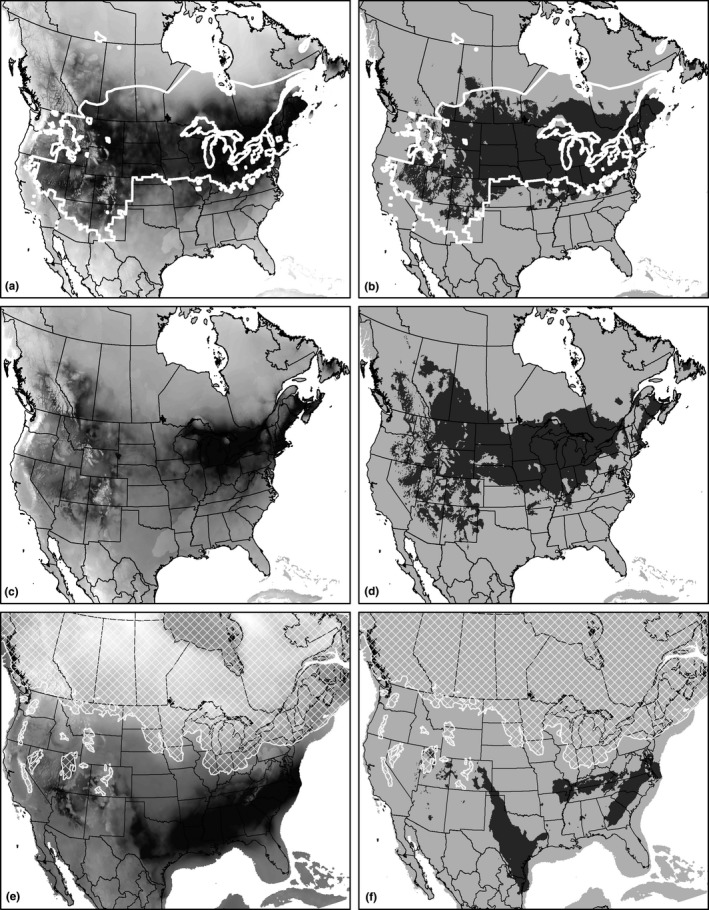
Maps of species distribution model results showing habitat suitability (darker is more suitable; a, c, e) and expected range (in dark gray; b, d, f). The maps for the present day (a, b) show the actual modern range of *Rana pipiens* outlined in white. The maps for the Last Glacial Maximum (21 kya; e, f) show the extent of the glaciers at that time in white hatching

The Mahalanobis distance and Maxent models were the species distribution models that performed best in predicting the occurrence of *R. pipiens* based on the k‐fold validation, with AUC scores ranging from 0.889 to 0.902, meaning the models correctly identified presence points in the validation dataset nearly 90% of the time. As a result, these were weighted more heavily than the BIOCLIM and logistic regression models when making the final predictions (Table 5). When comparing the climatic suitability and expected range of the species distribution model to the known range of the northern leopard frog (Hammerson et al. 2004), there is broad agreement. However, the species distribution model appears to slightly underestimate the expected range in the desert southwest of the United States and along the northern limits of the range in Canada (Figure 8a,b). The underestimate of *R. pipiens* range in the desert southwest of the United States could be due to unique local adaptations of populations in that region that were drowned out by the components of the climate niches occupied by other northern leopard frog populations (Valladares et al., 2014). It could also be a reflection of the modern climate being marginally suitable for *R. pipiens* in this region, as *R. pipiens* is often listed as imperiled in most of the states on the western and southern edges of its range. Finally, the underestimate of the northern edge of the range in Canada could be due to relatively fewer observations in the north, biasing the models toward the more heavily sampled core and southern areas of the northern leopard frog range.

Projection of the species distribution model onto the climate of the mid‐Holocene showed very similar suitability and expected range to the present day (Figure 8c,d). The only major differences include the contraction of the range in the Pacific northwest of the United States and Canada and an expansion in the northeastern United States and Canada from the mid‐Holocene to the present. The projection of the species distribution model onto the climate of the Last Glacial Maximum shows a range shifted further south, well below the glacial extent (Figure 8e,f). The expected range is split between parts of the American Atlantic Plain and areas around the Ohio River Valley, while most of the Great Plains region appears unsuitable, and areas stretching from eastern Colorado to the Gulf coast of Texas could have supported *R. pipiens* populations during this period.

## 4 DISCUSSION

### 4.1 Population structure, landscape effects, and prehistoric climate influences on population differentiation

There is strong evidence of population structuring of *R. pipiens* in North Dakota. The populations are primarily split into a western clade to the southwest of the Missouri River and an eastern clade to the north and east of the Missouri River. The eastern clade is structured into six distinct clusters, and the western clade is structured into four distinct clusters. These patterns of population structure were consistent across three structuring methods with different underlying mechanisms and demonstrate the strengths of using multiple clustering methods to analyze population genetic data. River basins and the Missouri River were the most important landscape features in determining the spatial pattern of population structuring, along with isolation by distance.

Acting as a barrier, the Missouri River has prevented gene flow between *R. pipiens* populations since the retreat of glaciers from North Dakota at the end of the Wisconsin glaciation. Both a partial Mantel test and the RDA variance partitioning (Table 3) identified the Missouri River as a statistically significant factor in the structuring of *R. pipiens* populations. Rivers are commonly identified as barriers to gene flow in other amphibians (Fouquet, Ledoux, Dubut, Noonan, & Scotti, 2012; Garcia, Ivy, & Fu, 2017; Li, Chen, Tu, & Fu, 2009). While clearly separating distinct populations, the clustering analyses also indicate some areas of the Missouri River may be slightly permeable. Sample sites 16 and 17 were located in wetlands connected to the east bank of the Missouri River and contain several individuals with genotypes that identify them as putative migrants (Q > 0.5 for a population in a different basin than sample was collected in) or descendants of migrants from populations west of the Missouri River (Q ~ 0.5 for two different populations; Figure 3). The Mississippi River plays a similar role acting as a strong, but slightly permeable barrier between the major evolutionary lineages of *R. pipiens* in eastern and western North America (Hoffman & Blouin, 2004a). The role the Missouri River has played in the evolutionary history of *R. pipiens *in North Dakota is not entirely clear. The area to the southwest of the Missouri River was the only glacier‐free area in North Dakota during the Last Glacial Maximum (Mickelson et al., 1983), but the bioclimatic modeling indicated that this region was not suitable during the glacial maximum. However, following glacial retreat, the area west of the Missouri was likely to be the first area colonized by *R. pipiens *within North Dakota. Subsequently, the Missouri River has allowed the *R. pipiens* populations that colonized the southwest portion of North Dakota to remain genetically distinct from the *R. pipiens* populations that colonized the region to the east of the Missouri River.

Hoffman and Blouin (2004a) identified a single refugium for the western lineage of *R. pipiens* located in southern Nebraska, while the eastern lineage of *R. pipiens* is likely descended from populations that occupied several refugia. The expected range for *R. pipiens* according to the species distribution model in this study largely agrees with that assessment, though Hoffman and Blouin (2004b) traced the east–west population divergence to a glaciation event prior to the Wisconsin glaciation during the Pleistocene. Still, the putative Pleistocene refugia in the species distribution model are geographically concordant with the putative refugia described in Hoffman and Blouin (2004a). Some areas of the expected range during the Pleistocene (Figure 8f) overlap the southern extent of the current range (Figure 8b) in the eastern United States. In the western United States, the northern edge of the expected suitable range is shifted far south into eastern Colorado. The western refugium identified by Hoffman and Blouin (2004a) in Nebraska is outside of the expected range defined by the species distribution model, but this could be explained by the pattern of slight underestimation for the northern edge of suitable climate for *R. pipiens*, or if the identified refugium populations were closely related to unsampled populations in the actual refugium areas in eastern Colorado, Oklahoma, or Texas.

The main split between *R. pipiens* on opposite sides of the Missouri River in North Dakota most likely occurred approximately 13–18 kya, during an expansion period out of the western refugium to the south following the retreat of glaciers. The colonization route these lineages followed into North Dakota is not clear. These lineages may have followed the Missouri River northward along independent colonization routes on different sides of the river from the western glacial refugium. Alternatively, *R. pipiens* could have moved northward from the glacial refugium to colonize the western bank of the Missouri River, and the eastern populations in North Dakota are descendants of *R. pipiens* that crossed the Missouri River at a permeable location in the northern Great Plains. More sampling along the Missouri River and into the putative refugium area centered in eastern Colorado would be needed to reconstruct the colonization routes *R. pipiens* followed out of the western refugium following the Last Glacial Maximum.

Most of the population subdivision associated with river basins is most likely to have occurred during the mid‐Holocene (11–6 kya), a period when the northern Great Plains region went through extreme drought cycles (Valero‐Garcés et al., 1997; Xia et al., 1997). These drought periods would have extremely restricted the breeding and overwintering aquatic habitats that *R. pipiens* require, possibly confining them to major riparian areas or areas with comparatively higher annual rainfall (i.e., the Turtle Mountain ecoregion; Bryce et al., 1998). In fact, river basins, which accounted for over 20% of the spatial genetic structure (Table 3), have been found to be an important factor in the genetic structuring of other frog species (Lind, Spinks, Fellers, & Shaffer, 2011; Murphy, Dezzani, Pilliod, & Sorfer, 2010). Hydrological structure can impact genetic structure of amphibians through several mechanisms. Amphibians commonly use riparian areas to disperse, making river systems important migration corridors that facilitate connectivity within amphibian metapopulations (Mullen, Arthur Woods, Schwartz, Sepulveda, & Lowe, 2010; Shephard & Burbrink 2011; Howell, Muths, Hossack, Sigafus, & Chandler, 2018; Murphy, Jones, Price, & Weisrock, 2018). Additionally, in the prairie pothole region, overwintering habitats impose the greatest constraints on the distribution of *R. pipiens* (Mushet 2010). During droughts, the permanent deepwater and flowing water habitats required for overwintering are clustered in low‐lying areas (Cohen et al., 2016; Van Meter & Basu, 2015). The connectivity of amphibian populations between basins is limited during dry periods when upland temporary wetlands are dry and *R. pipiens* are confined to overwintering sites in clusters of suitable wetlands in low‐lying areas of basins (Mushet et al., 2013). Recent work has shown landscape‐level decline of northern leopard frogs during severe droughts when populations are concentrated around the limited remaining winter refugia. Following the droughts, populations rapidly expanded (Mushet 2010). This interaction between drought and the hydrological structure of river basins in the northern Great Plains drives contraction–recolonization dynamics of *R. pipiens* populations and could explain the river basin‐associated pattern of genetic structuring seen in *R. pipiens* populations in North Dakota.

Isolated refugia during drought periods in the mid‐Holocene would explain the structuring and coalescence times seen in the *R. pipiens* populations in North Dakota. Reduced connectivity during long‐term droughts could also explain the most recent population divergence between *R. pipiens* in the Upper Red/Sheyenne and the Lower Red basins. The best support for this divergence in the DIY ABC model indicates it occurred approximately 600 years BP, during the Little Ice Age when the northern Great Plains had a relatively arid climate (Fritz et al., 1994; Laird, Fritz, Grimm, & Mueller, 1996; Xia et al., 1997), though the confidence intervals for this divergence also include other somewhat arid periods during the late Holocene. The dry period associated with the Little Ice Age lasted until approximately 200 years BP before shifting to the relatively wet current climate in eastern North Dakota (Fritz et al., 1994; Laird et al., 1996), a shift that likely restored connectivity and allowed the high amount of gene flow observed between the two subpopulations of *R. pipiens* located in the Red River basins.

Isolation by distance was the final landscape factor underlying the genetic variation of *R. pipiens* populations in North Dakota. Isolation by distance was strongly supported by Mantel tests (Figure 5) and was the most important single factor explaining the spatial genetic structure of *R. pipiens* in North Dakota according to the RDA variance partitioning analysis (Table 3). Isolation by distance is a common pattern seen in amphibian populations (Bani et al., 2015; Monsen & Blouin, 2004; Peterman, Feist, Semlitsch, & Eggert, 2013; Scribner, Arntzen, Cruddace, Oldham, & Burke, 2001), as even comparatively vagile amphibians like the northern leopard frog are dispersal limited (Hoffman, Schueler, & Blouin, 2004). *Rana pipiens* commonly disperse distances of 800 m from their natal ponds (Bartlet & Klaver, 2017; Knutson et al., 2018) and are capable of dispersing >5 km in some landscapes (Dole, 1971). *Rana pipiens* are more capable of long‐distance dispersal than many other amphibians, so patterns of isolation by distance may be difficult to detect in smaller areas with high wetland density due to high levels of gene flow (Mushet et al., 2013).

Land use did not appear to influence population structure of *R. pipiens* at the scale analyzed in this study. North Dakota has a fairly homogenous landscape, largely comprised of grassland, pasture, and cultivated crops. Intensive agriculture and cattle grazing can have negative effects on the dispersal ability of some amphibians (Mushet, Euliss, & Stockwell, 2012; Rothermel & Semlitsch, 2002; Vos, Goedhart, Lammertsma, & Spitzen‐Van der Sluijs, 2007). However, there is evidence that *R. pipiens* can use cultivated crop fields as well as native grasslands if the amount of cover and level of soil moisture in agricultural fields are comparable to the native grasslands allowing frogs to avoid desiccation (Bartlet & Klaver, 2017; Pope et al., 2000). Agricultural development may play a subtler role influencing connectivity within *R. pipiens* metapopulations than could be detected at the spatial scale used in this study. Finer scale sampling using higher‐resolution genetic markers may be able to detect local effects of land use on the biotic connectivity between prairie wetlands.

## 5 CONCLUSION

*Rana pipiens* populations in North Dakota are highly structured, typical of amphibian populations at regional geographic scales. The patterns of genetic population structure reflect the colonization history and historical climate influences of *R. pipiens* in the northern Great Plains region. The interplay between wetland connectivity and drought cycles appears to be one of the major factors influencing *R. pipiens* populations in this region. Wetland connectivity throughout North Dakota is currently being threatened by climate change, agricultural expansion, and wetland draining (Carter Johnson et al., 2010; McCauley, Anteau, Post van der Burg, & Wiltermuth, 2015). *Rana pipiens* populations southwest of the Missouri River, though currently stable, are characterized by lower genetic diversity (Stockwell et al., 2016) and smaller effective population sizes. These populations are at the highest risk of experiencing population declines similar to those reported on the western edge of the *R. pipiens* range. Conservation of wetlands and riparian areas across the region will be important, both for securing at risk *R. pipiens* populations on the Missouri Plateau and Badlands, and for maintaining the genetic diversity found throughout the *R. pipiens* populations on the glaciated northern Great Plains.

Any use of trade, firm, or product names is for descriptive purposes only and does not imply endorsement by the U.S. Government.

## CONFLICT OF INTEREST

None declared.

## AUTHOR CONTRIBUTIONS

CAS, JDLF, KP, and DM designed the project. JDLF performed fieldwork and laboratory work. KP and CAS provided guidance on data analyses. JW and JDLF conducted data analyses. CAS supervised the study. All authors participated in writing the manuscript.

## Data Availability

Sampling locations, microsatellite genotyping data, and R code for statistical analyses are available on Dryad, https://doi.org/10.5061/dryad.64s8h65.
