# Supplementary material for: Landscape genetics reveal broad and fine‐scale population structure due to landscape features and climate history in the northern leopard frog (Rana pipiens) in North Dakota
Source: Ecol Evol. 2019 Jan 15;9(3):1041–60. doi: 10.1002/ece3.4745 (PMC6374656; doi:10.1002/ece3.4745)
Supplement: Supplementary file 1 [file ECE3-9-1041-s001.pdf]

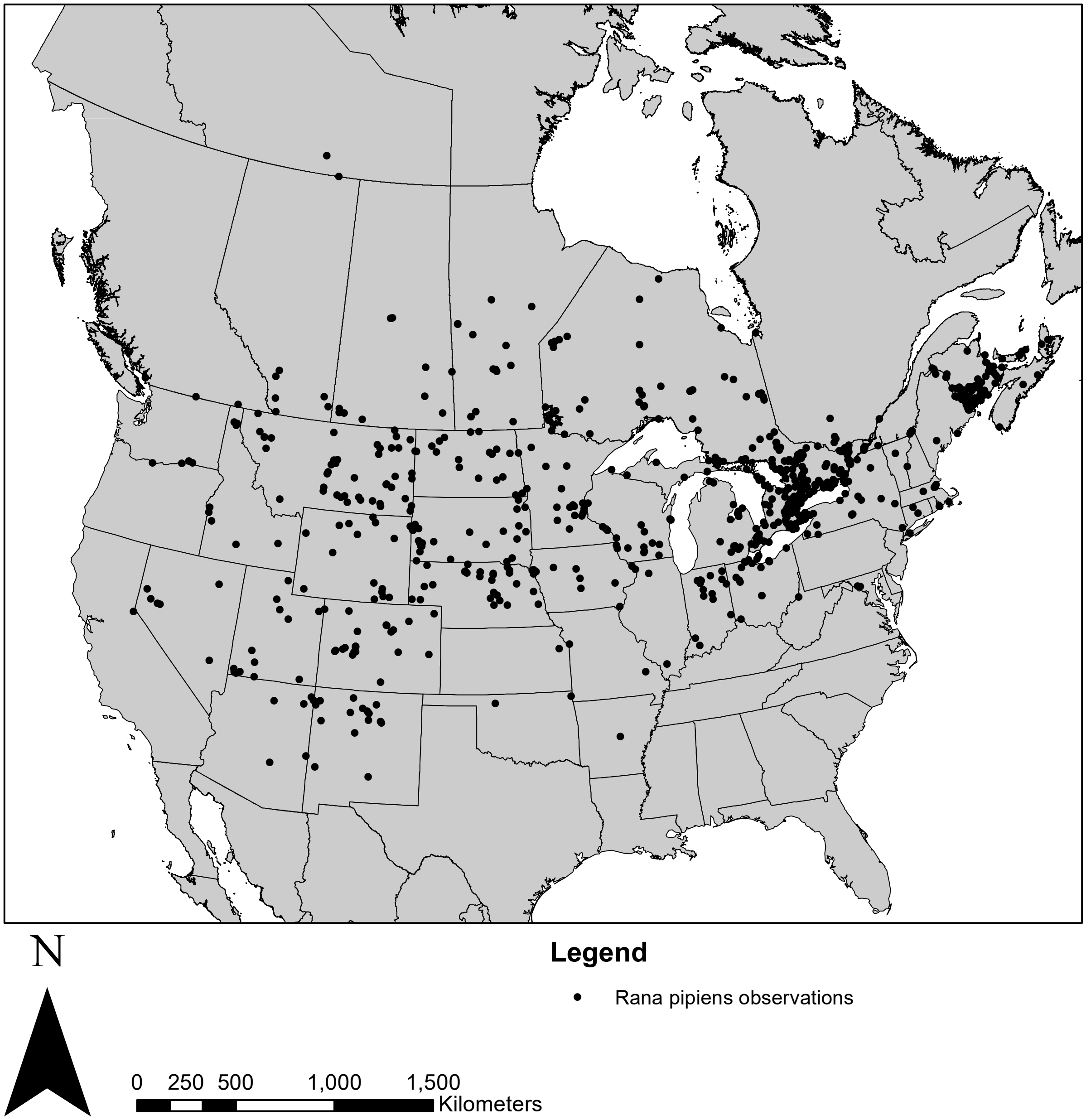

Supporting Information S1. Map of the 801 observation points of *Rana pipiens* from the VertNet database after filtering for erroneous records, duplicates, fossil records, and captive records. These were the presence points use to build the species distribution models.
